# Spawning seasons of Rasbora tawarensis (Pisces: Cyprinidae) in Lake Laut Tawar, Aceh Province, Indonesia

**DOI:** 10.1186/1477-7827-8-49

**Published:** 2010-05-18

**Authors:** ZA Muchlisin, Musri Musman, MN Siti Azizah

**Affiliations:** 1 School of Biological Sciences Universiti Sains Malaysia, Penang 11800, Malaysia; 2 Department of Aquaculture, Coordinator of Fishery and Marine Sciences, Syiah Kuala University, Banda Aceh 23111, Indonesia; 3 Centre for Marine and Coastal Studies Universiti Sains Malaysia, Malaysia; 4 Department of Marine Sciences, Coordinator of Fishery and Marine Sciences, Syiah Kuala University, Banda Aceh 23111, Indonesia

## Abstract

**Background:**

*Rasbora tawarensis* is an endemic freshwater fish in Lake Laut Tawar, Aceh Province, Indonesia. Unfortunately, its status is regarded as critical endangered with populations decreasing in recent years. To date no information on the spawning activities of the fish are available. Therefore, this study provides a contribution to the knowledge on reproductive biology of *R. tawarensis* especially on spawning seasons as well as basic information for conservation of the species.

**Methods:**

Monthly sampling was conducted from April 2008 to March 2009 by using selective gillnets. The gonadosomatic index, size composition and sex ratio were assessed. The gonadal development was evaluated based on macroscopic and microscopic examinations of the gonads.

**Results:**

The gonadosomatic index (GSI) varied between 6.65 to 18.16 in female and 4.94 to 8.56 for male. GSI of the female R. tawarensis was higher in March, September and December indicating the onset of reproductive seasons, the GSI and oocyte size being directly correlated with gonadal development stages. Although, a greater proportion of mature male than female was detected during the study, the sex ratio showed that the overall number of female was higher than male. The ovaries had multiple oocyte size classes at every stage of gonadal development, thus R. tawarensis can be classified as a group synchronous spawner or a fractional multiple spawner.

**Conclusion:**

The spawning seasons of *R. tawarensis* were three times a year and September being the peak of the reproductive season and the female was the predominant sex. This species is classified as a group synchronous spawner.

## Background

*Rasbora tawarensis *is an endemic freshwater fish in Lake Laut Tawar. It is one of the major freshwater fish, the main target for fishing in the lake and of considerable commercial importance in the region. It is listed in the IUCN red list as vulnerable [1] and updated by CBSG as critically endangered due to the very restricted area of its distribution [2]. Presently, the *R. tawarensis *population is decreasing, indicated by decreasing catch-per-unit effort (CPUE), where the average CPUE decreased from 1.17 kg/m^2 ^of net in the 1970s to only 0.02 kg/m^2 ^of net in 2009 (Muchlisin, unpublished data). Unfortunately, many basic life history characteristics of the *R. tawarensis *have not been documented.

Studies on reproductive behaviour of fish are important and a basic requirement for improvement and effective fishery resources management and conservation [3-7], determination of basic life-history information and for assessing the impacts of environmental variability on the dynamics of fish populations [8]. Life history parameters such as spawning frequency and sex ratio may vary between populations of a species and temporally within population [9]. Several studies describing various aspects of reproductive biology have been conducted for many freshwater tropical cyprinids for example the snakeskin gouramy, *Trichogaster pectoralis *[10], *Thynnichthys thynoides *[3], snakehead, *Channa striata *[11], rainbow selebensis, *Telmatherina celebensis *[12], bonti-bonti, *Paratherina striata *[13] and serandang, *Channa pleurophthalmus *[14] and However, to date there has been limited study done on *R. tawarensis *despite its enormous fishery and ecological importance. Hence, the objective of the present study was to evaluate the spawning periodicity and sex ratio of *R. tawarensis*.

## Methods

### Site and sampling technique

Lake Laut Tawar (04°36'43"N 096°55'25"E) is situated in Aceh Tengah, Aceh Province, Indonesia. It is located approximately 1,200 m above sea level. The lake is an old volcanic caldera of circa 16 km length, 5 km width and maximum predicted depth of 80 m. It is surrounded by mountains reaching over 2000 meters. At least 25 short tributaries discharge into Lake Laut Tawar, the main outflow is Peusangang River. The watershed is covered by forests, which are increasingly affected by deforestation and agricultural activities.

Samplings were conducted from April 2008 to March 2009 using selective gillnet (mesh size 5/9 inch, 1.5 m depth and 20 m length). The gill nets were set up for eleven hours (18.00 PM to 05.00 AM) and every sampling trip was for two days. Collected fishes were counted, rinsed and anesthetized in a solution of tricaine methanesulfonate (MS 222), prepared by dissolving 4 g of MS 222 in 5L tap water, then after preserved in 10% formalin in a plastic bag. The plastic bag was tagged by catching location, date and name of fish. The fish samples were transported to the laboratory for further evaluation.

### Gonadosomatic index (GSI) and gonadal development stages

Specimens were measured to the nearest mm (total length-TL and standard length-SL) using a digital balance (Toledo, AB-204. Error = 0.01 g), and weighed to the nearest gram by using a pair of digital callipers (Mitutoyo, CD-6CS. Error = 0.01 mm). The gonads were removed by abdominal dissection and weighed to the nearest gram. The GSI was calculated using the formula below [15,16]:

where GSI is gonadosomatic index, GW is gonad weight (g) and BW is total body weight (g) with intact gonad.

Gonad development stage was determined and classified based on macroscopic and microscopic characteristics of the gonad modified from West [17] and Marcano [18]; i.e. immature, developing, mature, ripe and spent. Representative gonads were randomly taken from each stage of gonadal development to measure diameter of oocytes. At least 50 oocyte samples from anterior, middle and posterior of the ovary were measured by using a stereo microscope CCD camera. The oocyte size was calculated by using the formula: length axis plus wide axis divided by two.

### Histological procedure

Samples of the central portion of the gonads of 0.5 cm thickness were washed, dehydrated in an increasing ethanol series, n-butilic alcohol, embedded in paraffin and sectioned for 7-10 μm in thickness using a microtome (Reichert-Jung 820, Germany). The sections were stretched in a water bath (40°C) of distilled water. Three replicate section samples were gathered with the object glass properly labelled and dried for 24 hours at 37°C followed by one hour at 60°C over a stove. Sections were stained with a solution of Ehrlich hematoxilline and eosine for a general assessment of the histological components of the gonads.

### Data analysis

The data were subjected to an analysis of variance (ANOVA), followed by comparison of means using Duncan's multiple range test to determine significance of each data treatment [19]. All statistical analyses were performed using SPSS v14.

The linear regression and correlation analyses were utilized to describe relationships between GSI and total length or body weight, GSI and proportion of mature female, while the power regression was performed to describe the relationship between spawning frequency and proportion of mature females.

## Results

### Gonadal development and spawning season

**Figure 1:**
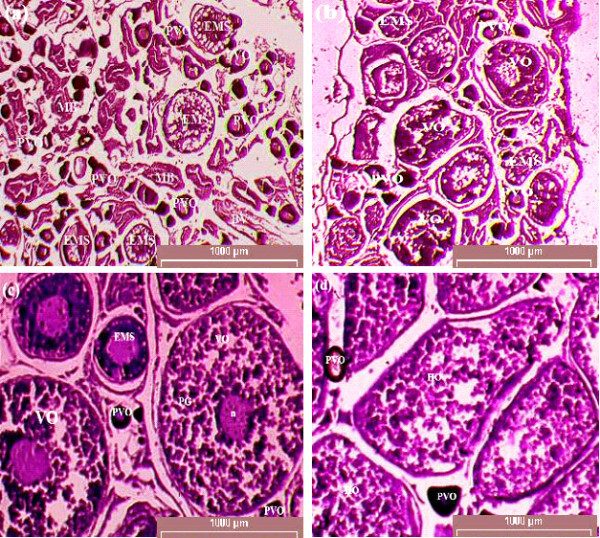
**(a) Immature female shows many oocytes have not yet developed, (b) developing female shows many oocytes are still growing at an early maturation stage (c) mature female, (d) post ovulatory female shows hydrated oocytes**. BV, blood vessel; EMS, early mature stage oocytes; HO, hydrated oocytes; MB, muscle bundle; PVO, previtellogenic oocytes; VO, vitellogenic oocytes; n, nucleus; PG, protein granule.

Based on macroscopic and microscopic evaluation, the gonadal development stages of *R. tawarensis *were divided into 5 classes (Table 1). A least two developmental stages of oocytes were present in the ovary (Figure 1). Fish with gonad stages III and IV were considered sexually mature. In general, all stages of gonads were observed in every monthly sampling, where a higher proportion of mature females were present in September (48.28%) and March (30.88%), but lower in April (2.08%). While, higher percentage of mature males was found in January and September but lower in August (Table 2). The oocyte sizes varied depending on gonadal development stages; Stage I (immature) 209.61 μm to 592.78 μm (447.30 ± 99.42 μm), Stage II (developing) 528.37 μm to 867.10 μm (711.24 ± 63.21 μm), Stage III (mature) between 604.15 μm to 894.33 μm (780.59 ± 50.71 μm), stage IV (ripe) between 725.27 μm to 991.81 μm (844.09 ± 57.59 μm) and stage V (spent) between 227.64 μm to 770.82 μm (447.38 ± 139.02 μm) (Table 1).

**Table 1:** Female and male gonad developmental stages in *R tawarensis *and their descriptions.

Stages	Classification	Macroscopic appearance		Oocyte size (μm)	GSI range	
		Testes	Ovary		F	M
I	Immature	Small, flat, translucent to whitish, poorly developed, with reduced fringes.	Small, transparent to translucent and not very voluminous. Oocyte not visible with naked eye.	209.61-592.78 (447.30 ± 99.42)^a^	<10.9	<2.0
II	Develop	Whitish with voluminous fringes.	Large orange-pale, oocytes may be visible through the ovary tunic.	528.37-867.10 (711.24 ± 63.21)^b^	11.0-18.9	2.5-5.5
III	Mature	Very large, firm, white in colour.	Very large occupying part of the abdominal cavity. Yellow oocyte turgescency.	604.15-894.33 (780.59 ± 50.71)^c^	19.0-23.9	5.6-8.0
IV	Ripe	Full developed, turgid fringes, milky-whitish in colour. Milt run out of the fish.	Occupying the entire abdominal cavity. Ovulated oocytes can be fully expelled from the oviduct with gentle pressure.	725.27-991.81 (844.09 ± 57.59)^d^	>24.0	> 8.0
V	Spent	Bloody and flaccid fringes.	Flaccid, red-brown or bloody in colour. Few remaining large oocytes observed, and smaller size oocytes may be seen.	227.64-770.82 (447.38 ± 139.02)^a^	2.5-10.9	1.5-3.0

*Mean oocyte size in the same column followed by a different superscript is significant different (P < 0.05).

**Table 2:** Proportion of gonadal development stages according to monthly sampling (I = Immature, III = Develop, III = Mature, IV = Ripe, V = Spent).

Month	No. of female	Stages of gonad maturity												No. of male
		Female (%)						Male (%)						
		I	II	III	IV	V	Mature III+IV	I	II	III	IV	V	Mature III+IV	
January	89	50.56	32.58	7.87	2.25	6.74	10.12	-	6.25	56.25	37.50	-	93.75	16
February	86	61.63	33.72	2.33	-	2.33	2.33	12.00	12.00	64.00	12.00	-	76.00	25
March	68	20.59	45.59	19.12	11.76	2.94	30.88	-	20.00	26.67	53.33	-	80.00	15
April	48	72.92	14.58	2.08	-	10.42	2.08	7.14	28.57	35.71	21.44	7.14	57.15	42
May	65	33.84	49.23	12.31	3.08	1.54	15.39	-	16.22	24.32	56.76	2.70	81.08	37
June	60	41.66	35.00	15.00	1.67	6.67	16.67	1.79	57.14	21.43	16.07	3.57	37.50	56
July	79	53.16	24.06	7.59	6.33	8.86	13.92	3.84	50.00	25.00	11.54	9.62	36.54	52
August	91	27.47	51.65	12.09	7.69	1.10	19.78	-	60.00	20.00	13.33	6.67	33.33	15
September	58	8.62	41.38	31.04	17.24	1.72	48.28	-	10.53	15.79	73.68	-	89.47	19
October	73	54.79	23.29	5.48	5.48	10.96	10.96	-	20.00	56.00	12.00	12.00	68.00	25
November	96	48.96	20.83	5.21	4.17	20.83	9.38	5.88	41.18	23.53	17.65	11.76	41.18	17
December	88	19.32	52.27	20.45	5.68	2.28	26.13	-	27.27	54.55	18.18	-	72.73	22

**Figure 2:**
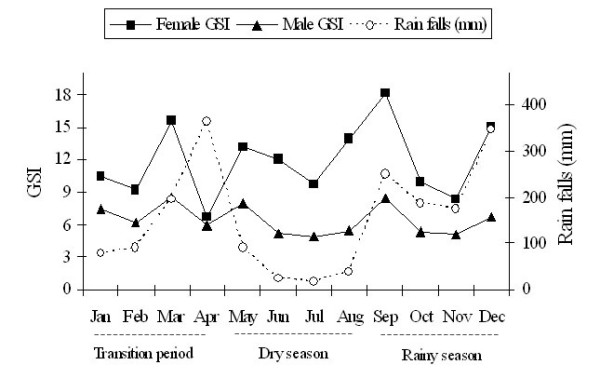
**The GSIs showing the spawning season of *R. tawarensis *in relation to rain fall and seasons**.

The monthly GSIs of female *R. tawarensis *varied from 6.65 in April to 18.16 in September and 4.94 to 8.56 in male (Table 3). Female GSI was consistently higher than in male. The GSI value for both sexes begin to rise from January to March followed by a steep fall in April, and again rise sharply from July with a second peak in September before declining to resting level in November and again reaches a third peak in December (Figure 2). According to a three-year meteorological data (2004 - 2006) highest rainfall (with long rainy days) occurred in April, September and December, lowering drastically in January and then steadily increasing in April followed by a dry season from May to August. This largely coincided with the spawning periods.

**Table 3:** Gonadosomatic Index (GSI) and sex ratio according to monthly sampling

Months	No. of fish examined for GSI		GSI		Sex ratio (F/M)
	Female	Male	Female	Male	
January	89	16	10.48 ± 6.18^bc^	7.39 ± 1.44^bcd^	5.56
February	86	25	9.21 ± 5.08^b^	6.16 ± 2.41^ab^	3.44
March	68	15	15.56 ± 6.88^f^	8.56 ± 2.53^d^	4.53
April	48	42	6.65 ± 4.94^a^	5.95 ± 2.46^ab^	1.14
May	65	37	13.24 ± 5.13^de^	7.91 ± 2.10^cd^	1.76
June	60	56	11.99 ± 5.83^cd^	5.22 ± 3.07^a^	1.07
July	79	52	9.70 ± 7.52^b^	4.94 ± 2.63^a^	1.52
August	91	15	13.95 ± 6.27^def^	5.46 ± 2.63^a^	6.07
September	58	19	18.16 ± 5.39^g^	8.44 ± 2.18^d^	3.05
October	73	25	9.96 ± 6.63^bc^	5.30 ± 3.97^a^	2.92
November	96	17	8.38 ± 7.12^ab^	5.14 ± 2.41^a^	5.65
December	88	22	15.08 ± 6.00^ef^	6.68 ± 1.82^abc^	4.00

*Mean of GSIs in the same column followed by a different superscript indicates significantly different (P < 0.05).

Female GSIs which peaked in September was significantly different from other months (P < 0.05), then followed by March, but however, was not significantly different from August and December (P > 0.05). Male GSIs peaked in March, but was not significantly different from September, May and January (P > 0.05). This indicated that the reproductive activity of *R. tawarensis *was highest in September.

### Size composition and sex ratio

The monthly sample data showed that females ranged between 67.43 mm to 109.55 mm (89.40 ± 5.17 mm) in total length and 2.43 g to 9.40 g (5.44 ± 0.95 g) in weight. Males were 64.82 mm to 98.84 mm (81.12 ± 5.12 mm) in total length and 2.25 g to 7.32 g (4.43 ± 0.85 g) in weight. In addition, based on all data (including data from additional sampling in July 2009), female ranged between 62.20 mm to 113.31 mm in size (89.39 ± 6.74 mm) and 2.43 g to 11.83 g weight (5.55 ± 1.14 g) while male was 55.22 mm to 102.47 mm (80.23 ± 8.43 mm) in length and 1.29 g to 8.75 g (4.49 ± 1.15 g) in weight. This indicated that the females were significantly larger than males.

**Figure 3:**
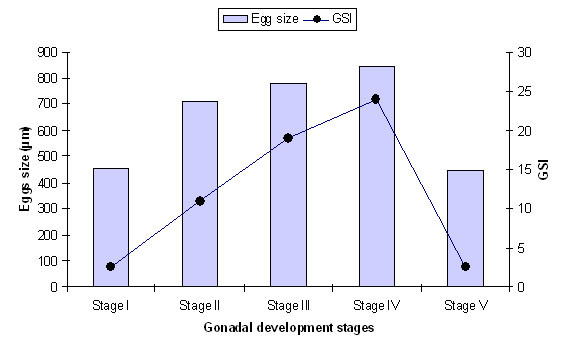
**A plot of egg size against GSI and gonadal developmental stage of *R. tawarensis***.

**Figure 4:**
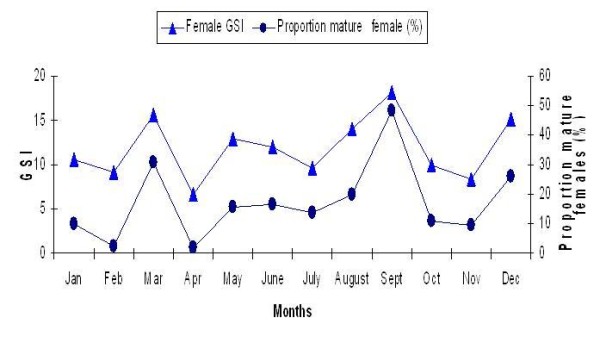
**A plot of relationship between GSI and proportion of matured female of *R. tawarensis***.

Mature females ranged from 70.98 mm to 113.31 mm in length and 3.52 g to 11.83 g in gonad-free body weight while males ranged from 58.06 mm to 102.474 mm in length and 2.71 g to 8.75 g in gonad-free body weight. In general, oocyte sizes and GSI increased from stage I to stage IV (immature to ripe stages) and then decreased in stage V (spent), being at the maximum during the period of peak reproductive season and declining abruptly thereafter, when the fish become spent (Figure 3). The GSI was correlated with the proportion of matured female, where GSIs increased with increasing proportion of mature fish (Figure 4).

**Figure 5:**
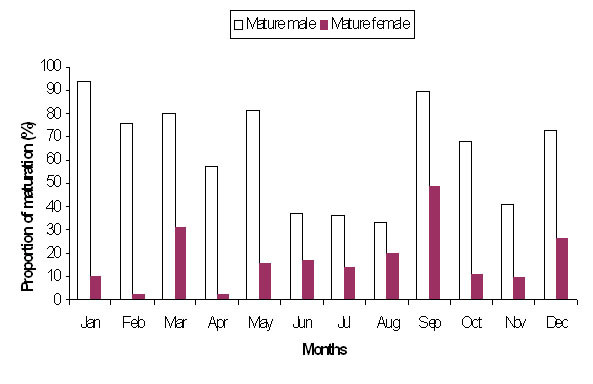
**Comparison of sexual maturation between male and female *R. tawarensis *throughout the year**.

The sex ratio (female: male) of *R. tawarensis *ranged from 1.07 in June to 6.07 in August (Table 3), with an average of 3.39 ± 1.8 indicating that females were dominant in the population. Although, less abundant in total, the proportion of matured male was consistently higher throughout the year (Figure 5).

## Discussion

The GSI values of *R. tawarensis *for both sexes with peaks in March, September and December, presumably the likely onset of the spawning seasons largely coincided with the rainy seasons. Our observation showed that the fish migrates from the lake to river tributaries for spawning, frequently during rainstorm in the rainy season. Its spawning season occurs twice during the rainy season i.e. early and at the end of the season. In addition during the transition period where the rain falls and the rainy days were relatively higher compared to the dry season, the fish is also triggered to spawn. According to Rainboth [20] the spawning activities of Southeast Asian cyprinids are accomplished in a variety of ways; longitudinal migration from downstream to up stream and vice versa or even laterally from the stream they inhabit into temporary flooded riparian areas or tributaries. Many studies have reported high correlation of rainy season with spawning peaks of tropical fishes associated with flooding of rivers and lakes, or the monsoons. For example *T. thynnoides *in the Chenderoh Reservoir, Malaysia spawned during the rainy season in January, August, and November when the water level was high [3]. The spawning season of African bonytongue fish in the So River in the floodplain of West Africa occurred during the wet season (May to August) as floodwaters gradually rose [21]. In addition, the reproduction in *Tor putitora *was observed mainly in the autumn months of March to April and also in the monsoon months, from July to August. During these months, *T. putitora *migrated from the main river to the tributaries where it bred in the flooded waters [22]. In general, fishes in the tropics depend on rainfall to trigger the reproductive cycle as the stable temperature and photoperiod could not generate reproductive cues [10].

Typically, fish migrate upstream to spawn when the water level increases during the rainy season, to ensure that the current brings eggs and larvae into nursery areas on the floodplain further downstream. During this season the fish feed intensively in the flood zone, growing and building up fat layers for the following dry season, when the food is scarce [23]. The periodic floods provide increased available habitat and also releases nutrients that evoke blooms of phytoplankton and an increase in micro zooplanktonic food organisms for the hatchling fishes [24]. The increased water level, inundation of shallow areas, increase in water velocity and turbidity may be responsible for inducing the fish to spawn [25] especially for fishes in the flood plain areas [26]. This is also the main feeding and growing period for many tropical fishes, when they build up fat stores to carry them through the dry season [24]. In addition, in tropical zones, seasonal changes of environment are less extreme, and many fishes exhibit extended or continuous reproductive pattern [27]. This is in agreement with the moderate levels of GSI in January and February as observed in this study. Thus these add further support that the rainy season or rainfall plays an important role in reproductive period of tropical fishes.

It is highly likely that *R. tawarensis *is capable of spawning throughout the year, as mature males and females were detected throughout the year, although more abundant during certain periods with a peak season in September. A similar phenomenon was observed in *C. pleurophthalmus *[14] and *Astyanax fasciatus *[28] which had the potency to spawn throughout the year, with spawning peak influenced by water temperature and rainfall. Based on gonadal development and variation of oocyte size in the ovary, *R. tawarensis *can be classified as a group synchronous spawner or a fractional multiple spawners, having two or more distinct clutches of oocyte existing concurrently with each clutch at a different developmental stage. According to Redding and Patino [27], this pattern allows for multiple, distinct ovulatory events that typically follow seasonal, lunar, or diurnal cycles.

Female GSI values of *R. tawarensis *were consistently higher than in male, a phenomenon also observed in the freshwater catfish *Oxydoras sifontesi *and *Pimelodus blochii *from Venezuelan floodplains [18] and *A. fasciatus *from south-eastern Brazil [28]. The GSI is one of the main parameters used to evaluate gonadal development in fishes and this method is easier and cheaper to utilize. The high correlation of GSI with number of matured females and males could be utilised to extrapolate peak spawning season. Furthermore, the GSI and length frequency distributions provide good population-level information of reproductive performance [29].

The sex ratio of *R. tawarensis *fluctuated seasonally. This is in agreement with Nikolsky [30] who reported that the sex ratio may vary from year to year in the same population, but in most fish species it is close to one, for example in the rainbow selebensis, *T. celebensis *[12], *Protopterus annectens *[31] and *Oreochromis niloticus *[32]. However, the sex ratio of *R. tawarensis *showed a predominance of female, a similar trend to that reported for *Tilapia mariae *[33], *A. fasciatus *[28] and *Pellonula leonensis *[34]. In contrast, in *Abudefduf saxatilis *[35] and *T. putitora *[22], the number of male was higher than female.

However, the reported sex ratios may have be biased due to selectivity of fishing gear, therefore independent data from other fishing gears would be required to validate whether the samples obtained from existing gears were representative of the population [7]. Furthermore, the seasonal variation in the sex ratio observed was probably because once fertilization of eggs was completed, male possibly emigrates from spawning area towards feeding ground located in the shallow areas [32].

## Conclusions

Gonadosomatic indices (GSI) of female *R. tawarensis *were highest in the months of March, September and December with the peak in September, indicating the onset of the reproductive seasons. However, matured fishes were detected throughout the year. The *R. tawarensis *was classified as a group synchronous spawner. In addition, the female was predominant in the population.

## Competing interests

The authors declare that they have no competing interests.

## Authors' contributions

ZAM, the author responsible for developing of the study design, data collection and analyses, data interpretation and manuscript drafting. MM, the author responsible for data collection, statistical analysis, and intellectual contents. SAMN, the author responsible for manuscript sequence alignment, language corrections and final approval of the manuscript to publish. All authors read and approved the final manuscript.
